# miR-34a Inhibits Migration and Invasion of Tongue Squamous Cell Carcinoma via Targeting MMP9 and MMP14

**DOI:** 10.1371/journal.pone.0108435

**Published:** 2014-09-30

**Authors:** Ling-fei Jia, Su-bi Wei, Keith Mitchelson, Yan Gao, Yun-fei Zheng, Zhen Meng, Ye-hua Gan, Guang-yan Yu

**Affiliations:** 1 Department of Oral and Maxillofacial Surgery, Peking University School and Hospital of Stomatology, Beijing, China; 2 Medical Systems Biology Research Center, Tsinghua University, Beijing, China; 3 Department of Oral Pathology, Peking University School and Hospital of Stomatology, Beijing, China; 4 Central Laboratory, Peking University School and Hospital of Stomatology, Beijing, China; 5 CapitalBio Corporation, Changping District, Beijing, China; University of Barcelona, Spain

## Abstract

**Background:**

miR-34a is an important tumor suppressor gene in various cancer types. But little is known about the dysregulation of miR-34a in tongue squamous cell carcinoma (TSCC). In this study, we investigate the expression and potential role of miR-34a in TSCC.

**Methods:**

We evaluated miR-34a expression and its relationship with clinicopathological characters in 75 pairs of TSCC samples, and confirmed the role of miR-34a for predicting lymph node metastases from a further 15 pairs of paraffin-embedded TSCC specimens with stringent clinicopathological recruitment criteria using quantitative reverse transcription polymerase chain reaction (qRT-PCR). The effects of miR-34a on cell proliferation, migration and invasion were examined in TSCC cell lines using Cell Counting Kit-8 assay, wound healing assay and transwell assay, respectively. The effects of miR-34a on the expression of matrix metalloproteinase (MMP) 9 and 14 were detected by luciferase reporter assays and Western blot analysis. The expression of miR-34a, MMP9 and MMP14 were also confirmed in TSCC samples by *in situ* hybridization and immunohistochemistry.

**Results:**

miR-34a expression in tumor tissues from TSCC patients with positive lymph node metastases was significantly lower than that with negative lymph node metastases. Overexpression of miR-34a significantly suppressed migration and invasion in TSCC cells and simultaneously inhibited the expression of MMP9 and MMP14 through targeting the coding region and the 3′untranslated region, respectively. Moreover, miR-34a expression in TSCC was inversely correlated with protein expression of MMP9 and MMP14 in the TSCC samples.

**Conclusions:**

miR-34a plays an important role in lymph node metastases of TSCC through targeting MMP9 and MMP14 and may have potential applications in prognosis prediction and gene therapy for lymph node metastases of TSCC patients.

## Introduction

Tongue squamous cell carcinoma (TSCC) is the most common type of oral cancer and is characterized by its high rate of proliferation and lymph nodal metastases [1], [2]. The presence of cervical lymph node metastases is one of the most important prognostic factors for patients with TSCC [3], [4]. In clinical practice, if cervical lymph node metastases of TSCC are apparent at presentation of patients, neck dissection is necessary. However, the treatment of early-stage TSCC patients with clinically negative cervical lymph node is still controversial [5]. Although clinicopathological characteristics often guide the clinician's treatment choices, biomarkers of cervical lymph node metastases in TSCC would greatly assist the decision-making for appropriate clinical treatment [6]. Therefore, understanding the molecular pathways of TSCC lymph nodal metastases in TSCC would be helpful in improving diagnosis, and potentially therapy of this disease.

MicroRNAs (miRNAs) are endogenously expressed small non-coding RNAs of 19–24 nucleotides (nt) that regulate gene expression by either inhibiting translation or by inducing degradation of their target messenger RNAs (mRNAs) [7], [8]. Several miRNAs, such as miR-184/138/21/195, have been shown to have the critical roles in the development and progression of TSCC [9], [10], [11], [12]. miR-34a is a well recognized tumor suppressor gene in various cancer types, in which it can inhibit cell proliferation, induce cell apoptosis and senescence by targeting to CDK4/6, Cyclin E2, Cyclin D1, E2F3, Bcl-2, MYCN, Notch1/2 and SIRT1 [13], [14], [15], [16], and can inhibit cell migration and invasion by targeting c-Met, Notch1, Jagged1 and Fra-1 [14], [17], [18], [19]. Although a previous study demonstrated that miR-34a has anti-angiogenic functions in head and neck squamous cell carcinoma (HNSCC) [20], the relationship between the expression of miR-34a and lymph node metastases of TSCC patients remains to be investigated and whether there are other targets of miR-34a that regulate cancer cell migration and invasion needs to be elucidated. Matrix metalloproteinases (MMPs) are secreted during the growth, invasion, metastases, and angiogenesis of tumors, and can affect the surrounding microenvironment, causing dynamic changes of bio-behavior of the tumor [21]. However, the precise molecular mechanism relationships between miR-34a and MMPs in the cellular malignant and invasive phenotypes are not fully understood.

Here, we reported that miR-34a expression in tumor tissues from TSCC patients with positive lymph node metastases was significantly lower than that with negative lymph node metastases. Mechanistic analysis showed that miR-34a inhibited migration and invasion of TSCC cell lines via targeting the coding region of MMP9 and the 3′untranslated region (UTR) of MMP14.

## Materials and Methods

### Ethics Statement

These experiments were approved by the Institutional Ethics Committee of Peking University School of Stomatology (Approval number PKUSSIRB-2012010) and all samples were obtained from patients who signed informed consent forms approving the use of their tissues for research purposes after surgery.

### Human tissue specimens

All tissue specimens were collected from the Department of Oral and Maxillofacial Surgery, Peking University School of Stomatology. For freshly frozen tissues, paired primary TSCC samples from anterior portions of the tongue and adjacent nonmalignant tissues that were at least 1.5 cm distal to the tumor margins were obtained from 75 TSCC patients who underwent operations between May 2008 and August 2011. These freshly frozen specimens were also used in our previous study [12]. The median duration of follow-up was 25 months (range, 9–48 months). Tumor tissues and the matched nonmalignant tissues were snap-frozen in liquid nitrogen and then stored at −80°C until use. For clinical stage, information obtained from clinical examination and radiologic imaging was used to stratify the patients into I-IV clinical stage (cTNM). For lymph node metastases, the pathologic stage (pTNM) information derived from histopathologic examination of the regional lymph nodes was used to judge the patients lymph node metastases status. The clinicopathological characteristics of patients were summarized in Table 1.

**Table 1:** Correlation between miR-34a expression and clinicopathologic parameters in 75 TSCC patients.

Characteristics	No.	miR-34a (T/N)	*P*
Sex			0.744
Male	41	0.771±0.349	
Female	34	0.747±0.295	
Age			0.771
<60 y	41	0.770±0.316	
≥60 y	34	0.748±0.337	
Tumor size			**0.030**
T_1_–T_2_	49	0.819±0.348	
T_3_–T_4_	26	0.649±0.240	
Differentiation			0.838
Well	33	0.784±0.332	
Moderate	35	0.737±0.304	
Poor	7	0.765±0.415	
Clinical stage			0.881
I-II	44	0.765±0.286	
III-IV	31	0.753±0.375	
Node metastases			**0.001**
Yes	40	0.649±0.296	
No	35	0.887±0.311	
Final outcome			**0.030**
Survival	44	0.828±0.351	
Death	31	0.664±0.256	

Abbreviations: T, tumor; N, nonmalignant tissue; T_1_–T_4_: T stage of TNM classification system.

The formalin-fixed paraffin-embedded (FFPE) tissues were obtained from 30 patients who were selected from amongst about 1,000 TSCC patients who had undergone operations at the hospital between May 2000 and October 2007. The inclusion criteria were: the clinical data showed that the carcinoma was located in the body (anterior portions) of the tongue, clinical negative cervical lymph node (cN0), no distant metastasis (M0), tumor size limited in T2 and T3. Histological examination showed moderate differentiation. The patients received removal of the primary carcinoma and neck dissection without preoperative radiotherapy or chemotherapy, and were followed-up for at least 5 years post-operation. These 30 patients were divided into 2 groups according to the histological examination and the results of follow-up. One group of 15 patients called the “fortunate group”: histological examination of the operative specimen did not show cervical lymph node metastasis, all of the patients were alive for more than 5 years after operation. The second group of 15 patients was called the “unfortunate group”, histological examination showed cervical lymph node metastasis, all of the patients died within 4 years after operation. Age and gender were also matched individually between the 2 groups (Table S1).

For both freshly frozen and FFPE samples, tumor size and clinical stage were classified according to the TNM staging system [22] and each of the surgical samples we examined was confirmed by the pathologists using H & E-stained sections.

### RNA isolation and quantitative reverse transcription PCR (qRT-PCR)

For freshly frozen tissues, total RNA from tumor and normal tissue samples was isolated by using TRIzol reagent (Invitrogen, Carlsbad, CA) according to the manufacturer's instructions. For each of the FFPE samples, total RNA was isolated from five sections (10 µm thickness/section) using miRNeasy for FFPE kit (Qiagen, Valencia, CA) according to the manufacturer's instructions. The expression of miR-34a was determined by quantitative stem-loop reverse transcription qRT-PCR [23]. Primers for qRT-PCR of miR-34a, MMP9, MMP14,U6 (internal control for miRNAs) and β-actin (internal control for mRNAs) are listed in Table S1. Quantitative PCR was conducted at 95°C for 10 min followed by 40 cycles of 95°C for 15 sec and 60°C for 60 sec in an ABI 7500 real-time PCR system. The relative expression levels of miR-34a, MMP9 and MMP14 were calculated using the 2^−△△Ct^ method.

### Construction of expression vectors and RNA oligoribonucleotide

The empty vector, pcDNA3.0, and a miR-34a expression vector, pcDNA3.0-miR-34a, were gifts from Professor Moshe Oren (Weizmann Institute of Science, Israel) [24]. Similar to a previous study, the miR-34a inhibitor (anti-miR-34a), with a sequence complementary to miR-34a, and the anti-miR-NC used as a negative control for anti-miR-34a were synthesized by Genepharma (Shanghai, China) [25]. Human MMP9 and MMP14 full-length cDNA cloned into pcDNA3.0 vector (Invitrogen, Carlsbad, CA) was purchased from Integrated Biotech Solutions Company (Ibsbio, Co., Ltd., Shanghai, China) and named as pcDNA3.0-MMP9 and pcDNA3.0-MMP14. The small interfering RNAs (siRNAs) targeting human MMP9 and MMP14 transcripts named as siMMP9-1, siMMP9-2, siMMP14-1, and siMMP14-2, respectively, and siRNA control were all purchased from Integrated Biotech Solutions Company (Ibsbio, Shanghai, China). Sequences of miR-34a inhibitor and the siRNAs were listed in Table S2.

### Cell cultures and transfection

Primary normal human oral keratinocyte (HOK) cells were purchased from ScienCell Research Laboratories (San Diego, CA, USA) and were cultured in a keratinocyte growth medium (ScienCell Research Laboratories Inc.) following the manufacturer's instructions. Human tongue cancer cell lines SCC-15 and CAL27 were purchased from American Type Culture Collection (ATCC, Manassas, VA, USA), cultured as described previously [12]. Cells were seeded onto 6-well plates the day before transfection to ensure 80% confluence at the time of transfection. Transfection with 4 µg of expression vectors or 50 nM siRNAs was performed using Lipofectamine 2000 (Invitrogen) in accordance with the manufacturer's instrction. One well was used to assess the equal efficiency of transfection with an empty pcDNA3.0 vector containing the gene for green fluorescent protein (GFP) or 5′carboxyfluorescein (FAM)-labeled oligonucleotides (Ibsbio, Co., Ltd.), from which we consistently observed >80% transfection efficiency.

### Establishment of miR-34a stable overexpressing transfectants

The empty lentivirus vector contains the gene for GFP, pLL3.7, and a miR-34a expression lentivirus vector, pLL3.7-miR-34a were purchased from Integrated Biotech Solutions Company (Ibsbio, Co., Ltd.). Vesicular stomatitis virus G envelope protein pseudotyped viruses were prepared by packaging the retroviral or lentiviral vectors in 293T cells by transient transfection using the calcium phosphate method, as previously described [26]. After infection of CAL27 cells with either pLL3.7 or pLL3.7-miR-34a, the infected cells were screened and sorted by a FACS based on the expression of GFP which indicated the presence of the plasmids. The sorted pLL3.7 or pLL3.7-miR-34a infected CAL27 cells were then used as the stable NC or miR-34a overexpression model as previously described [27] (Figure S1). We referred to the infected cells as either NC or miR-34a groups respectively, in the following experiments.

### Cell proliferation assay

The effects of miRNA-34a overexpression and deregulation of MMP9 or MMP14 on cell proliferation were assessed using the Cell Counting Kit-8 (CCK-8, Dojindo, Kumamoto, Japan). Briefly, SCC-15 and CAL27 cells were seeded onto 96-well plates (2×10^3^ cells/well) and transfected with pcDNA3.0 (NC) and pcDNA3.0-miR-34a (miR-34a) as indicated. CCK-8 (10 µl) was added to each well at various time points and incubated at 37°C for 3 h. The absorbance at 450 nm was measured using a microplate spectrophotometer (Bio-Tek Instruments Inc, Winosski, VT).

### Wound healing assay

SCC-15 and CAL27 cells were cultured in a 6-well culture plate and transfected with miR-34a, siMMP9 and siMMP14 using Lipofectamine 2000. Subsequently, wounds were created in the confluent cells using a 200 µl pipette tip. The debris was removed by washing with serum-free medium. After 24 h of incubation, the cells that migrated into the wounded area or protruded from the border of the wound were photographed under an inverted microscope. Wound healing was quantified by measurement of the average linear speed of movement of the wound edges. Each experiment was independently performed at least three times.

### Transwell assay

The 8-µm pore size membrane, plain (for migration) or matrigel-coated (for invasion), transwell inserts (Costar, High Wycombe, UK) were placed in the wells of 24-well culture plates. 600 ul DMEM containing 10% fetal bovine serum was added to the lower chamber. SCC-15 and CAL27 cells were resuspended in 100 µl serum-free DMEM (1×10^5^ cells) and added to the upper chamber. After 24 h of incubation at 37°C with 5% CO_2_, cells on the topside of the filter were manually removed with a cotton swab. Cells adherent to the undersurface of the filter were fixed in cold methanol for 10 min and then stained with 0.01% crystal violet in 20% ethanol. After 10 min of incubation, the filters were washed thoroughly in water and images were taken and counted (5 random 200× fields per well).

### miRNA target validation

Predicted miR-34a target genes and their target binding sites regions were investigated using the RNA22 software, which does not need validated targets for training, and neither requires nor relies on cross-species conservation, and thus is an ideal tool for determining miRNA targets sites beyond the 3′UTR [28]. The potential binding sites of miR-34a from MMP1 to MMP14 were pedictied by RNA22 software. The results showed that only MMP9 (GenBank accession number, NM_004994.2) and MMP14 (GenBank accession number, NM_004995.3) mRNA contain putative miR-34a target sites. The miR-34a target sequences in the coding region of MMP9 were amplified by PCR and then cloned into a modified version of pcDNA3.1(+) that contained a firefly luciferase reporter gene (gift from Brigid L.M. Hogan, Duke University, Durham, NC, USA) [29], at a position downstream of a luciferase open reading frame similar to the method previously described [30]. The fragment of 3′UTR of human MMP14, which contained predicted target site of miR-34a, was amplified by PCR and cloned into the downstream of the modified pcDNA3.1(+) luciferase reporter, between the *Eco*RI and *Xho*I cloning sites. These vectors were named wild type coding region or 3′UTR. The site-directed mutagenesis of the miR-34a binding site in MMP9 coding region and MMP14 3′UTR were performed using Site-Directed Mutagenesis Kit (SBS Genetech, Beijing, China) and named as mutant coding region or mutant 3′UTR. All constructs were confirmed by DNA sequencing. CAL27 cells grown in a 48-well plate were co-transfected with 400 ng of either pcDNA3.0 or pcDNA3.0-miR-34a, 40 ng of the firefly luciferase reporter plasmid including the wild type or mutant 3′UTR of MMP9 or MMP14, and 4 ng of pRL-TK, a plasmid expressing Renilla luciferase (Promega, Madison, WI). Luciferase activity was measured 24 h after transfection using the Dual-Luciferase Reporter Assay System (Promega).

### Western blot analysis

Western blot was performed as described previously [12]. The primary antibodies against MMP9 (Cell Signaling Technology, Beverly, MA), MMP14 (Abgent, San Diego, CA), Cyclin D1 (Santa Cruz Biotechnology, Santa Cruz, CA),CDK6 (Cell Signaling Technology), Bcl-2 and β-actin (Santa Cruz) were diluted 1∶1,000. To quantify the intensity of the bands obtained through Western blot assay, ImageJ software-based analysis (http://rsb.info.nih.gov/ij/) was applied. The background was subtracted, and the signals of the detected bands were normalized to that of loading control β-actin bands.

### Immunohistochemistry and in situ hybridization

Immunohistochemical staining was performed with a two-step detection kit (Zhongshan Goldenbridge, Beijing, China) as previously described [12]. The primary antibodies were MMP9 (Cell Signaling Technology, 1∶150 dilution) and MMP14 (Abgent, 1∶100 dilution). The intensity of MMP9 and MMP14 immunoreactions was scored as follows: score 0, negative; score 1, weak; score 2 moderate; score 3, strong. miRNAs in situ hybridization assay were performed essentially as previously described [12]. Dual-DIG-labelled LNA probes miR-34a detection probe or Scramble-miR (negative control) were obtained from Exiqon (Exiqon, Vedbaek, Denmark) and the hybridizations were performed at 42°C. The probe sequences are listed in Table S1.

### Cell cycle and apoptosis analysis

At 48 h post-transfection, TSCC cells were harvested by trypsinization and washed with phosphate-buffered saline (PBS). Then, the cell cycle and apoptosis analysis were performed as described previously [12].

### Statistical analysis

All statistical analyses were performed using SPSS for Windows version 16.0. Student's *t* test and one-way ANOVA were used to analyze the relationship between miR-34a expression and clinicopathological characteristics. Correlation between miR-34a expression and MMP9 or MMP14 protein levels was analyzed using Spearman's rank correlation coefficient analysis with r and *P* values as indicated. Experiments with cell cultures were done at least in triplicate. Data were expressed as mean ± standard deviation (SD). A two-tailed value of *P*<0.05 was considered to be statistically significant.

## Results

### miR-34a expression was reduced in TSCC and correlated with clinicopathological features

**Figure 1:**
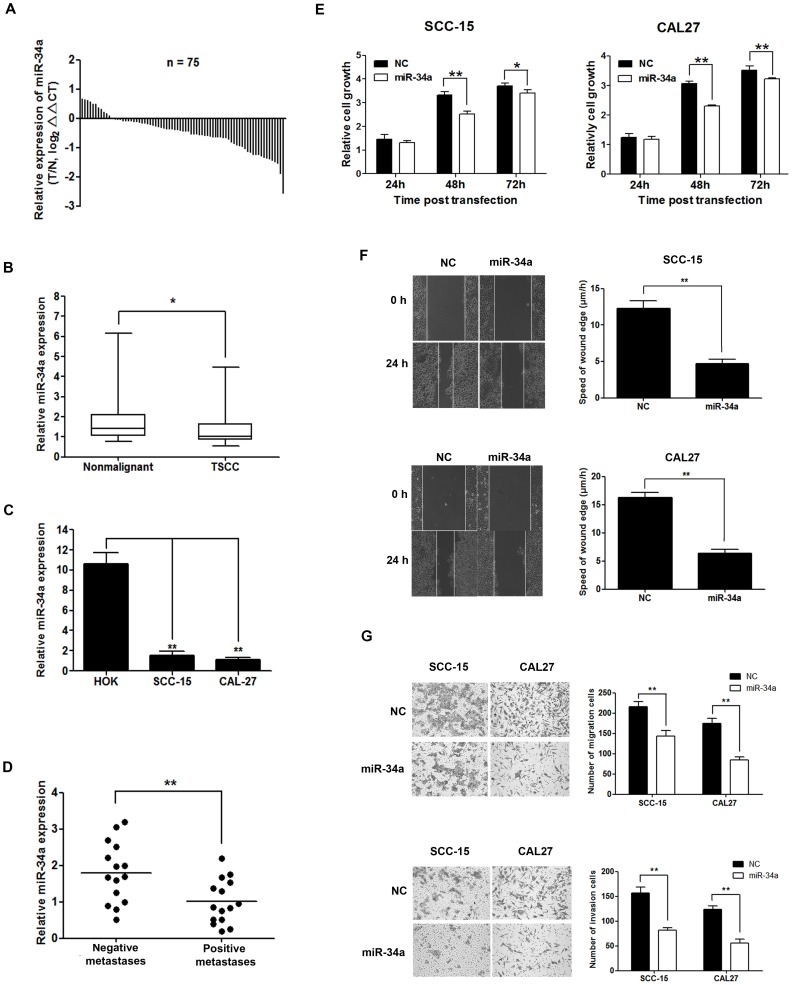
miR-34a correlated with lymph node metastases of TSCC patients and inhibited migration and invasion of TSCC cell lines. (A), Relative levels of miR-34a in 75 surgical specimens of TSCC and matched adjacent nonmalignant tissues was quantified by qRT-PCR. Data were presented as log2 fold change(△△Ct values,TSCC/Nonmalignant, T/N). (B), Means of miR-34a relative levels for 75 surgical specimens of TSCC and the matched adjacent nonmalignant tissues. Data were presented as 2^−△Ct (miR-34a-U6)^ values (*P<0.05). (C), Means of miR-34a relative levels in SCC-15, CAL27 cell lines and primary normal human oral keratinocyte (HOK) cells. Data were presented as 2^−△Ct (miR-34a-U6)^ values (**P<0.01). (D), Means of miR-34a relative levels from formalin-fixed paraffin-embedded tissues including a group of 15 TSCC patients with positive lymph node metastases compared with another group of 15 TSCC patients with negative lymph node metastases matched for age, gender, pathologic differentiation, and TNM stage. Data were presented as 2^−△Ct (miR-34a-U6)^ values (**P<0.01). (E), Cell viability was measured using CCK-8 assays, the data were presented as means ±SD as indicated (*P<0.05, **P<0.01). (F) and (G) Representative photomicrographs of wound healing and transwell assays results for SCC-15 and CAL27 cells transfected with pcDNA3.0-miR-34a (miR-34a) or pcDNA3.0 (NC) (×200 magnification, **P<0.01).

Expression of miR-34a was reduced in 63 of 75 (84%) tumor samples compared with their nonmalignant counterparts (Figure 1A). The average expression level of miR-34a was decreased significantly in tumor tissues compared with the nonmalignant tissues (Figure 1B). Moreover, miR-34a was also reduced significantly in the TSCC cell lines SCC-15 and CAL27, compared with HOK cells (Figure 1C). By normalizing miR-34a expression levels in the tumor tissues with those in adjacent nonmalignant tissues (Tumor/Nonmalignant, T/N), we observed statistically significant relationships between miR-34a expression (T/N) and several clinicopathological features of TSCC (Table 1), including tumor size (*P* = 0.030), node metastases (*P*<0.001) and patient mortality (*P* = 0.030).

### Decreased miR-34a expression was significantly correlated with lymph node metastases

We further analyzed the expression levels of miR-34a from FFPE tissues including a group of 15 TSCC patients with negative lymph node metastases (“fortunate group”) compared with another group of 15 TSCC patients with positive lymph node metastases (“unfortunate group”) matched for age, gender, location of the primary carcinoma, pathologic differentiation, TNM stage, and treatment modality (Table S2). The average expression level of miR-34a was statistically significantly decreased in the group with positive node metastases compared with that of the group with negative node metastases (Figure 1D, *P* = 0.005).

### Decreased miR-34a expression was significantly correlated with the survival of the patients

As shown in Table S2, 30 patients were divided into 2 groups according to their prognosis. One group had favorable prognosis with survival of at least 5 years (“fortunate group”) and another group had worse prognosis with death within 4 years (“unfortunate group”). The expression level of miR-34a in the “unfortunate group” was significantly lower than that in the “fortunate group” (Figure 1D, *P* = 0.005).

### miR-34a inhibited migration and invasion of TSCC cells

To investigate the effects of miR-34a on cell migration and invasion, miR-34a was transfected in SCC-15 and CAL27. After transfection, miR-34a expression was increased in both cell lines (Figure S2). Since ectopic expression of miR-34a in HNSCC cell lines is known to significantly inhibit tumor cell proliferation [20], and therefore may also affect migration and invasion assays. We investigated whether overexpression of miR-34a could inhibit cell proliferation of the two TSCC cell lines. Overexpression of miR-34a inhibited the viability of SCC-15 and CAL27 cells (Figure 1E), induced substantial accumulation of the cell population at the G1 stage of the cell cycle and promoted apoptosis in both cell lines at 48 h after transfection (Figure S3A and B). In addition, the endogenous expression of Cyclin D1, CDK6 and Bcl-2 proteins were significantly decreased in TSCC cells in which miR-34a was overexpressed (Figure S4). However, there was no significant difference in proliferation between miR-34a-transfected cells and the control at 24 h post-transfection (Figure 1E). We then performed cell migration and invasion assays within 24 h after transfection of miR-34a. The wound healing assay showed that overexpression of miR-34a significantly inhibited migration of TSCC cells compared with the negative control (Figure 1F). Similarly, both transwell migration and transwell invasion assays also demonstrated that migration and invasion of TSCC cell lines were inhibited by miR-34a overexpression (Figure 1G). Moreover, we used SCC-15 cell line, which showed a relatively higher level of miR-34a than CAL27 cells, to further evaluate the effects of miR-34a inhibition. The results showed that silencing of miR-34a by transfection of miR-34a inhibitor into SCC-15 cells increased their migration and invasion (Figure S5).

### miR-34a inhibited expression of MMP9 and MMP14 through targeting the coding region and 3′UTR, respectively

**Figure 2:**
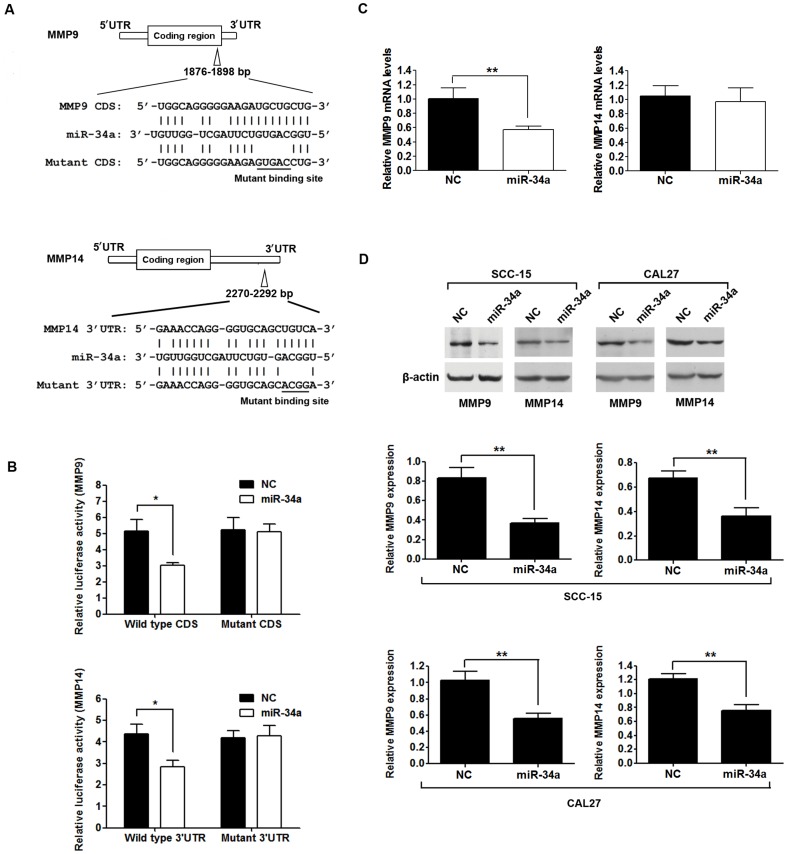
miR-34a targets MMP9 and MMP14. (A), The sequence of miR-34a (middle) matches the coding sequence (CD) of MMP9 and 3′untranslated region (UTR) of MMP14 (top). Bottom, mutations of the CD of MMP9 and 3′UTR of MMP14. (B), miR-34a inhibited wild-type, but not mutated MMP9 CD and MMP14 3′UTR luciferase reporter activity. CAL27 cells were co-transfected with firefly luciferase reporter plasmids containing wild type or mutant MMP9 CD and MMP14 3′UTR, and pRL-TK plasmid (a plasmid expressing rellina luciferase) and pcDNA3.0-miR-34a (miR-34a) or pcDNA3.0 (NC) as indicated. After 24 h, firefly luciferase activities were measured and normalized by use of renilla luciferase activities. Data were presented as mean ±SD (* *P*<0.05). (C), The relative MMP9 and MMP14 mRNA levels determined by qRT-PCR in miR-34a or NC transfected CAL27 cells (* *P*<0.01). (D), Inhibition of the expression of MMP9 and MMP14 proteins. Representative Western blotting image (top) and the quantification (bottom) of MMP9 and MMP14 proteins in miR-34a transfected SCC-15 and CAL27 cells. β-actin was used as internal control and was also detected by Western blot (** *P*<0.01).

According to predictions by RNA22 software, MMP9 mRNA contains a putative miR-34a target site in the coding region (1876–1898 nt) and MMP14 mRNA also a putative miR-34a target sites in the 3′UTR region (2270–2292 nt) (Figure 2A). We constructed luciferase reporter plasmids to contain the putative sequences for MMP9 or MMP14 or their corresponding mutant sequences as controls. Overexpression of miR-34a significantly suppressed luciferase activity of the reporter containing the miR-34a targeted wildtype sequences of MMP9 or MMP14, but not their corresponding mutant sequences (Figure 2B). Moreover, overexpression of miR-34a inhibited endogenous MMP9 mRNA expression (*P*<0.01), but not MMP14 mRNA expression (*P*>0.05) (Figure 2C). The protein levels of MMP9 and MMP14 were both reduced by miR-34a in SCC-15 and CAL27 cell lines (Figure 2D). Furthermore, after transfection of miR-34a inhibitor into SCC-15 cells, the protein levels of MMP9 and MMP14 were significantly increased (Figure S6). These results suggested that MMP9 and MMP14 could be direct targets of miR-34a.

### Knockdown of endogenous MMP9 or MMP14 inhibited migration and invasion of the TSCC cell lines

**Figure 3:**
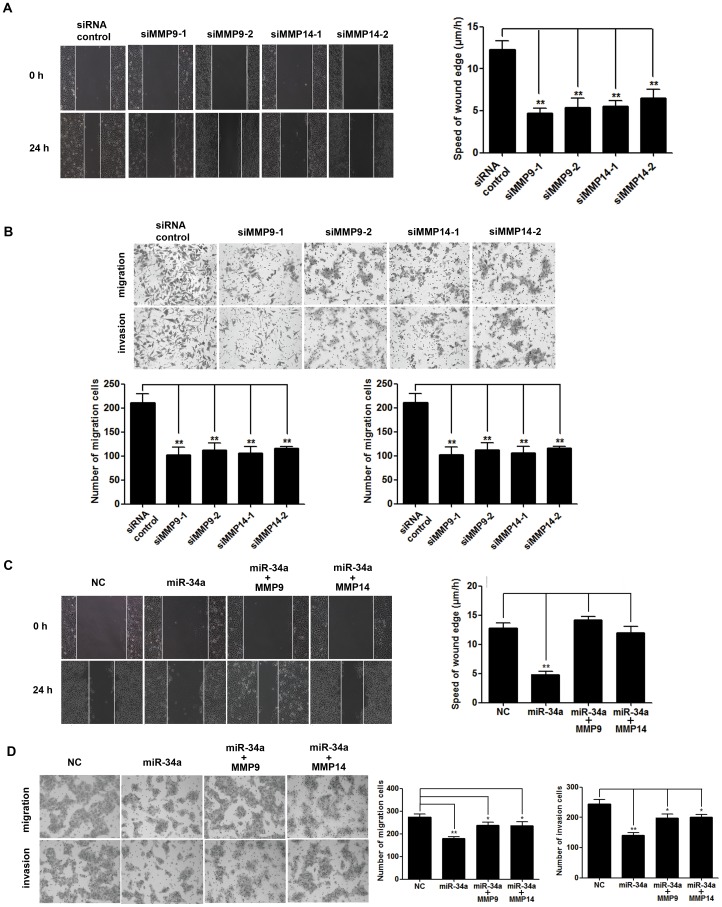
Inhibition of MMP9 and MMP14 was responsible for the TSCC cells migration and invasion effects of miR-34a. (A) and (B), Inhibition of cell migration and invasion by knockdown of MMP9 and MMP14. CAL27 cells were transfected with siRNA control, siMMP9-1, siMMP9-2, siMMP14-1, and siMMP14-2 as indicated. (C) and (D), MMP9 and MMP14 overexpression partially rescues miR-34a-reduced cell migration and invasion. Representative photomicrographs of wound healing and transwell assays results for stable expression miR-34a CAL27 cells transfected with pcDNA3.0-MMP9 (MMP9) or pcDNA3.0-MMP14 (MMP14) (×200 magnification, ** *P*<0.01).

To confirm whether downregulation of MMP9 and MMP14 by miR-34a could result in inhibition of migration and invasion of TSCC cells, we knocked down the expression of endogenous MMP9 or MMP14 by their siRNAs to mimic the effects of miR-34a overexpression. When the protein levels of both MMP9 and MMP14 were significantly reduced by siRNAs in CAL27 cell (Figure S7), migration and invasion of the cells were correspondingly significantly inhibited (Figure 3A and 3B), suggesting that the inhibitory effects of miR-34a on cells migration and invasion could, at least partially, act through its inhibition of MMP9 and MMP14 activities.

### Overexpression of MMP9 or MMP14 partially reversed inhibitory effects of miR-34a on TSC migration and invasion

Moreover, we used CAL27 cells stably transfected with miR-34a to test whether overexpression of MMP9 or MMP14 could reverse the inhibitory effects of miR-34a on migration and invasion of TSCC cells. The expression of miR-34a was significantly increased in the miR-34a stably-transfected cells (miR-34a) compared to cells stably-transfected with control vector (). As shown in Figure 3C and 3D, overexpression of MMP9 or MMP14 partially rescued migration and invasion capacities of CAL27 cells stably transfected with miR-34a.

### Expression of MMP9 and MMP14 proteins inversely correlated with miR-34a expression in TSCC

**Figure 4:**
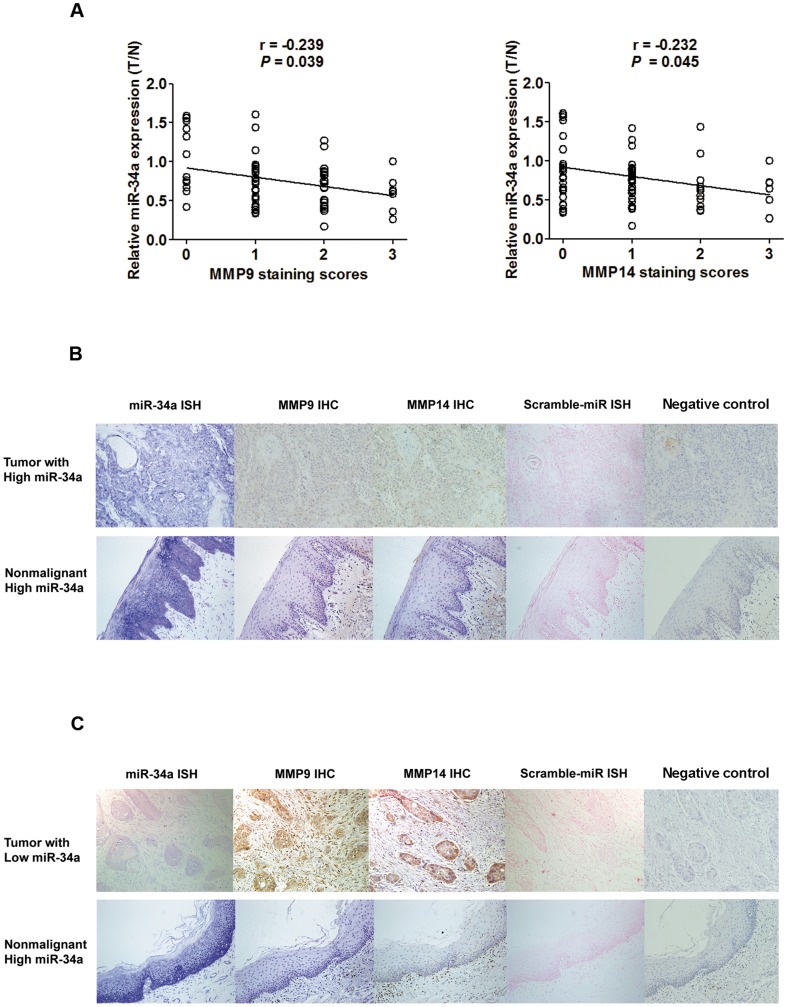
Inverse correlation between miR-34a and MMP9 or MMP14 protein levels in TSCC. The expression of MMP9 or MMP14 proteins was examined by immunohistochemistry (IHC) and miR-34a expression was detected by qRT-PCR and *in situ* hybridization (ISH). (A), Statistical analysis of the expression of miR-34a in tumor *vs.* nonmalignant tissue. Spearman's rank correlation analysis was performed, with r and *P* values as indicated. (B), The concurrence of high miR-34a expression and corresponding low expression of MMP9 or MMP14 were confirmed in TSCC and nonmalignant specimens by ISH with miR-34a detection probe or Scramble-miR and IHC, Primary antibodies were omitted in negative control for IHC (200× magnification). (C), The concurrence of low miR-34a expression and corresponding high expression of MMP9 or MMP14 was confirmed in TSCC and high miR-34a expression and corresponding low expression of MMP9 or MMP14 were confirmed in the matched nonmalignant specimens by ISH with miR-34a detection probe or Scramble-miR and IHC. Primary antibodies were omitted in negative control for IHC (200× magnification).

Since MMP9 and MMP14 transcripts were identified as direct targets of miR-34a, we examined the relationship between their protein expression and miR-34a expression in the paraffin sections of 75 TSCC samples and their matched nonmalignant samples using immunohistochemistry. Immunostaining of MMP9 and MMP14 in the tumor tissues were both inversely correlated with miR-34a expression measured by qRT-PCR (Figure 4A). Moreover, we also examined miR-34a expression in TSCC and matched nonmalignant tissues in consecutive sections using *in situ* hybridization. Both the immunohistochemistry and the *in situ* hybridization analysis showed that miR-34a expression was inversely correlated with immunostaining intensity of MMP9 and MMP14 in all TSCC specimens examined. Moreover, the immunostaining of MMP9 and MMP14 in TSCC adjacent nonmalignant tissues was generally of reduced intensity, coincident with the relatively high miR-34a expression (Figure 4B and 4C).

## Discussion

The present study demonstrated that the reduced expression of miR-34a in freshly frozen TSCC specimens was significantly correlated with tumor size, lymph node metastases and patients mortality. Among the three clinical factors, lymph node metastases of TSCC patients had the most significant statistical correlation with miR-34a expression. Then, we designed a case-control study in 30 TSCC FFPE samples to exclude the influence of some clinical factors, such as age, gender, location of the tumor, pathologic differentiation, TNM stage, and treatment modalities, which may influence the metastasis rate of cervical lymph nodes and the prognosis of the patients [31]. According to the pathological results on cervical lymph nodes and prognosis of the these patients, the patients were divided into a “fortunate group” and an “unfortunate group”. All the clinicopathological data on above mentioned factors influencing cervical lymph node metastasis and patient's prognosis were strictly controlled case by case. The results showed that the expression level of miR-34a in the “unfortunate group” with positive cervical lymph node metastasis was significantly lower than that in the “fortunate group” with negative cervical lymph node metastasis. These results provided further evidence that reduced expression of miR-34a is closely related to metastasis rate of cervical lymph nodes and prognosis of TSCC patients. More importantly, all the 30 FFPE TSCC samples were selected from the TSCC patients with the clinical node-negative (cN0) neck. In clinical practice, whether or not neck dissection should be performed to the TSCC patients with cN0 neck is still controversial [32], [33]. Currently, it is also difficult to accurately assess the metastatic status of the cervical lymph node [5]. Our results showed that miR-34a expression was significantly correlated to lymph node metastases of cN0 TSCC patients. Thus, the level of expression of miR-34a detected from TSCC biopsy samples could provide useful information for clinicians to make a choice whether or not neck dissection is performed.

As a potential functional explanation for miR-34a effects in lymph node metastases of TSCC, we demonstrated that miR-34a inhibited migration and invasion of TSCC cells and the results were consistent with a previous study [20]. Although cell migration and invasion occur as normal events in a number of physiological processes, uncontrolled migration and invasion are two key elements lead to metastases, which causes as high as 90% of human cancer deaths [31], [34]. Furthermore, we investigated the mechanism of the inhibitory effects of miR-34a on TSCC cells migration and invasion. MMP9 and MMP14, which have been regarded as regulators of tumor migration and invasion, are known to be involved in cell migration and invasion and frequently found to be upregulated in HNSCC [5], [33]. We performed a series of experiments using one TSCC cell line (CAL27) to demonstrate that the inhibition of MMP9 and MMP14 by miR-34a may at least partially account for the suppressing migration and invasion effect of miR-34a in TSCC cells.

Moreover, miR-34a targeted MMP9 at its coding region. In animals, miRNAs usually inhibit mRNA translation and decrease mRNA stability by binding sequences in the 3′UTR [35]. However, MMP9 transcripts have short 3′UTR (∼250 nt), miRNA recognition elements that can hardly be predicted in this restricted space. To the best of our knowledge, there is only one previous report showing that MMP9 can be regulated directly by miR-218 in osteosarcoma [36]. On the other hand, genes with shorter 3′UTRs have significantly more miRNA recognition elements in the coding region [37]. Our results showed that the predicted miR-34a target region of MMP9 has the potential to interact with 18 nt of miR-34a and miR-34a could regulate MMP9 through targeting at its coding region. Several studies have demonstrated that miRNAs can down-regulate their target genes via direct targeting the coding regions in animals [30], [38], [39]. However, the mechanism by which miRNAs mediate this manner of repression is not completely understood. Standart et al. suggested that miRNAs prevent the “closed loop” mRNA configuration induced by interaction of poly(A)-binding proteins with initiation factors at the 5′cap [40]. The predicted target region of miR-34a in MMP9 is located just around 240 nt before the stop codon, which is near the 3′UTR and the poly (A) tail. Therefore, it might allow for miRNA-mediated repression in a similar way as 3′UTR binding miRNAs. In addition, our results show that miR-34a directly down-regulated MMP9 that seems different from other studies, in which miR-34a indirectly down-regulates MMP9 expression through suppression of Fra-1 in colon cancer [18] and DLL1 in choriocarcinoma [41]. It appears that miR-34a may down-regulate MMP9 expression through both direct regulatory mechanism and indirect trans-regulatory mechanism. The current study also demonstrated that miR-34a down-regulated MMP9 by targeting the its 3′UTR. Besides, MMP14 has also been shown to be a direct target of miR-10b in glioma [42], miR-9 in neuroblastoma [43] and miR-133a in esophageal cancer [44]. These studies revealed that multiple miRNAs probably contribute to the loss of MMP14 expression in specific types of cancers.

Our *in vitro* experiments with human tongue cancer cell line SCC-15 and CAL27 also showed that miR-34a induced the G1-phase arrest and promoted cell apoptosis. Cyclin D1 and CDK6 are two of the key proteins involved in cell cycle control and are essential for G1 to S transition [45]. Bcl-2 is also one of the key regulators of cell apoptosis [46]. The present study confirmed that the protein expression of Cyclin D1, CDK6 and Bcl-2 were reduced by overexpression of miR-34a, which is consistent with previous studies in non-small cell lung cancer A549 cells [47] and neuroblastoma NLF cells [48]. These data suggested the potential tumor suppressor roles of miR-34a in TSCC.

In conclusion, our results demonstrated that miR-34a expression was significantly correlated with lymph node metastasis and prognosis of TSCC patients and could inhibit migration and invasion of TSCC cell lines via targeting MMP9 and MMP14. Overexpression of miR-34a in TSCC cells can also inhibit cell cycle progression and promoted cell apoptosis. The present study suggests that miR-34a have potential applications in prognosis prediction and gene therapy for lymph node metastases of TSCC patients.

## Supporting Information

Figure S1 **The transduction efficiency of the sorted lentivirus vectors pLL3.7 or pLL3.7-miR-34a infected CAL27 cells were>90% (100× magnification).**
(TIF)

Figure S2 **The expression of miR-34a was significantly increased in SCC-15 and CAL27 cells after transfection with pcDNA3.0 -miR-34a (miR-34a) and compared to transfection with pcDNA3.0 as negative control (NC).** Data was presented as mean ±SD (**P<0.01).
(TIF)

Figure S3 **Overexpression of miR-34a inhibited cell cycle progression and promoted cell apoptosis.** (A), Inhibition of cell cycle progression by overexpression of miR-34a. SCC-15 and CAL27 cells were transfected with pcDNA3.0, a negative control (NC) or with pcDNA3.0-miR-34a (miR-34a), as indicated. Cells were stained with propidium iodide (PI) at 48 h post-transfection and analyzed with FACS (** *P*<0.01). (B), Promotion of apoptosis by overexpression of miR-34a. SCC-15 or CAL27 cells were transfected for 48 h as in (A) and apoptotic cells were monitored with FACS after Annexin V and PI staining (** *P*<0.01).
(TIF)

Figure S4 **Overexpression of miR-34a decreased the endogenous protein expression of Cyclin D1, CDK6 and Bcl-2 in TSCC cell lines.** SCC-15 and CAL27 cells were transfected with pcDNA3.0 as a negative control (NC) or with pcDNA3.0-miR-34a (miR-34a) as indicated. After 48 h, Cyclin D1, CDK6 and Bcl-2 and internal control β-actin were detected by Western blot.
(TIF)

Figure S5 **Inhibition of miR-34a in SCC-15 significantly increased cell migration and invasion.** Representative photomicrographs of wound healing and transwell assays results for SCC-15 cells transfected with miR-34a inhibitor (anti-miR-34a) or the negative control (anti-miR-NC) (×200 magnification, * *P*<0.05).
(TIF)

Figure S6 **Inhibition of miR-34a in SCC-15 significantly increased protein levels of MMP9 and MMP14.** SCC-15 and CAL27 cells were transfected with miR-34a inhibitor (anti-miR-34a) or the negative control (anti-miR-NC) as indicated. After 48 h, MMP9, MMP14 and internal control β-actin were detected by Western blot.
(TIF)

Figure S7 **Inhibition of the expression of MMP9 and MMP14 by siRNAs targeting MMP9 and MMP14 transcripts.** CAL27 cells were transfected with siRNA control, siMMP9-1, siMMP9-2, siMMP14-1, and siMMP14-2 as indicated. After 24 h, MMP9, MMP14 and internal control β-actin were detected by Western blot.
(TIF)

Figure S8 **The expression of miR-34a was significantly increased in the miR-34a stably-transfected cells (miR-34a) compared to that stably-transfected with control vectors (NC).** Data was presented as mean ±SD (** *P*<0.01).
(TIF)

Table S1 **The clinical features of TSCC patients with positive and negative lymph node metastases.**
(DOC)

Table S2 **Sequences of RNA and DNA Oligonucleotides.**
(DOC)
