# Supplementary figures and images for: miR-34a Inhibits Migration and Invasion of Tongue Squamous Cell Carcinoma via Targeting MMP9 and MMP14
Source: PLoS One. 2014 Sep 30;9(9):e108435. doi: 10.1371/journal.pone.0108435 (PMC4182478; doi:10.1371/journal.pone.0108435)

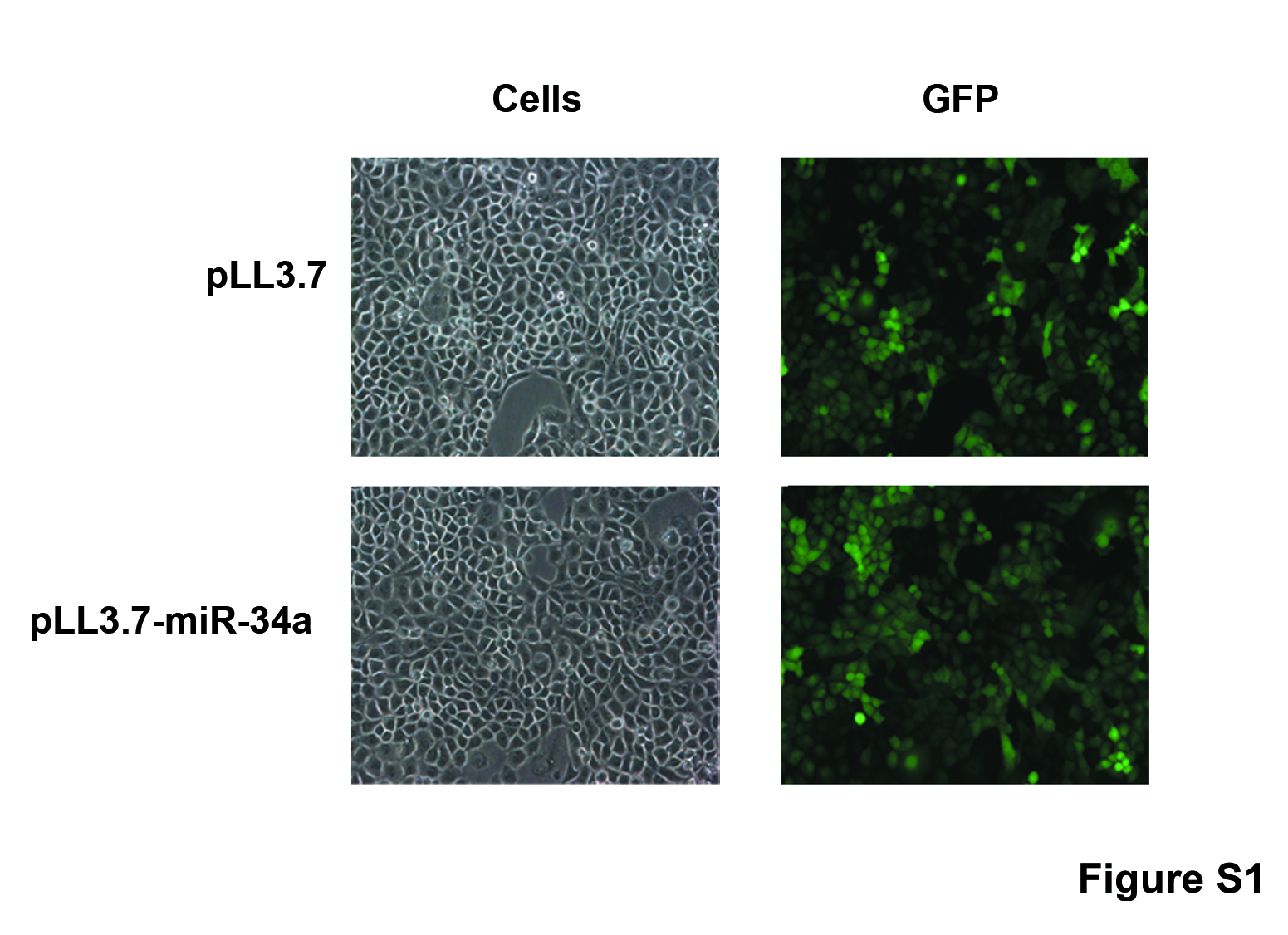

Supplement: Figure S1 — The transduction efficiency of the sorted lentivirus vectors pLL3.7 or pLL3.7-miR-34a infected CAL27 cells were>90% (100× magnification). (TIF) [file pone.0108435.s001.tif]

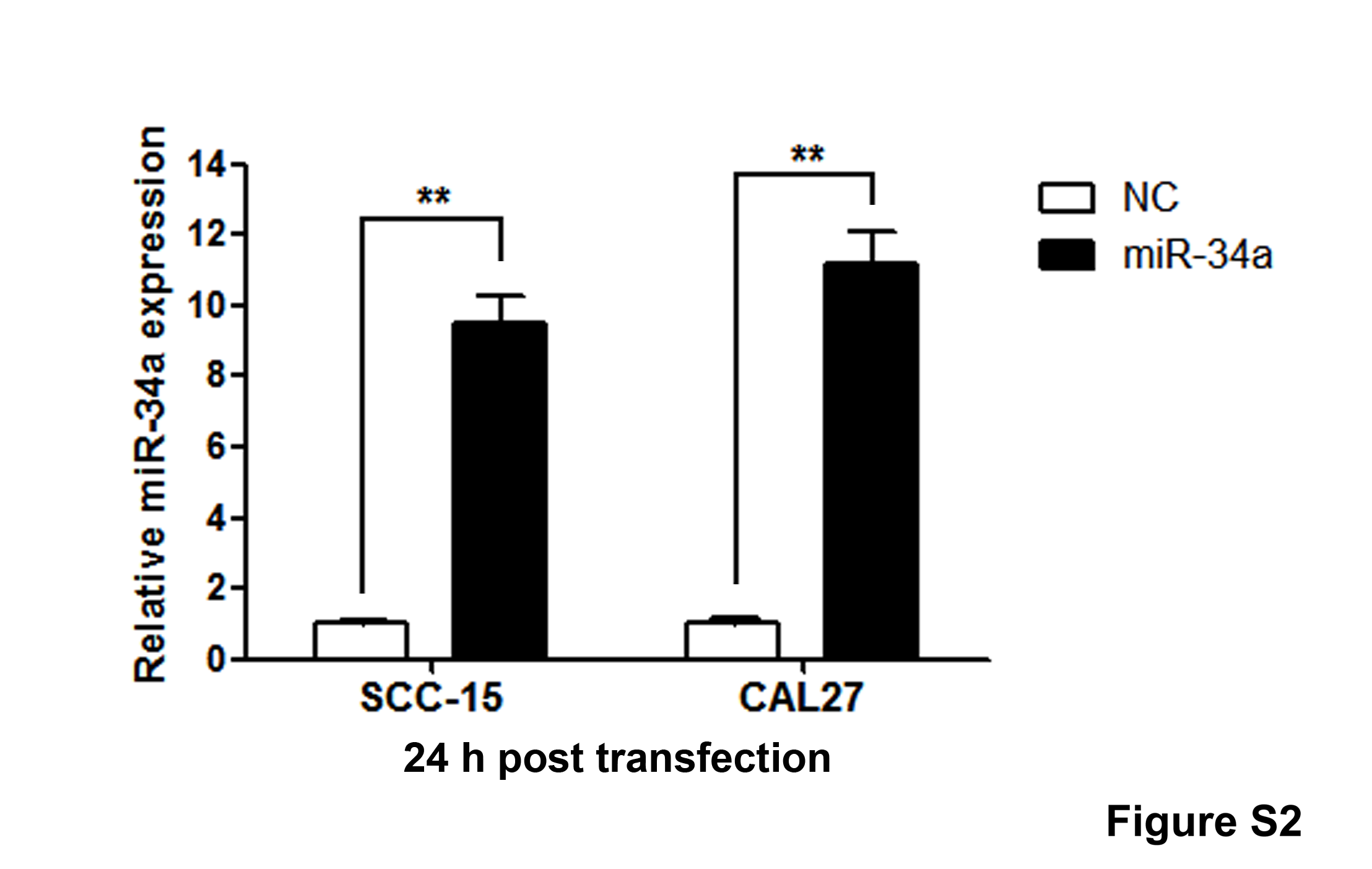

Supplement: Figure S2 — The expression of miR-34a was significantly increased in SCC-15 and CAL27 cells after transfection with pcDNA3.0 -miR-34a (miR-34a) and compared to transfection with pcDNA3.0 as negative control (NC). Data was presented as mean ±SD (**P<0.01). (TIF) [file pone.0108435.s002.tif]

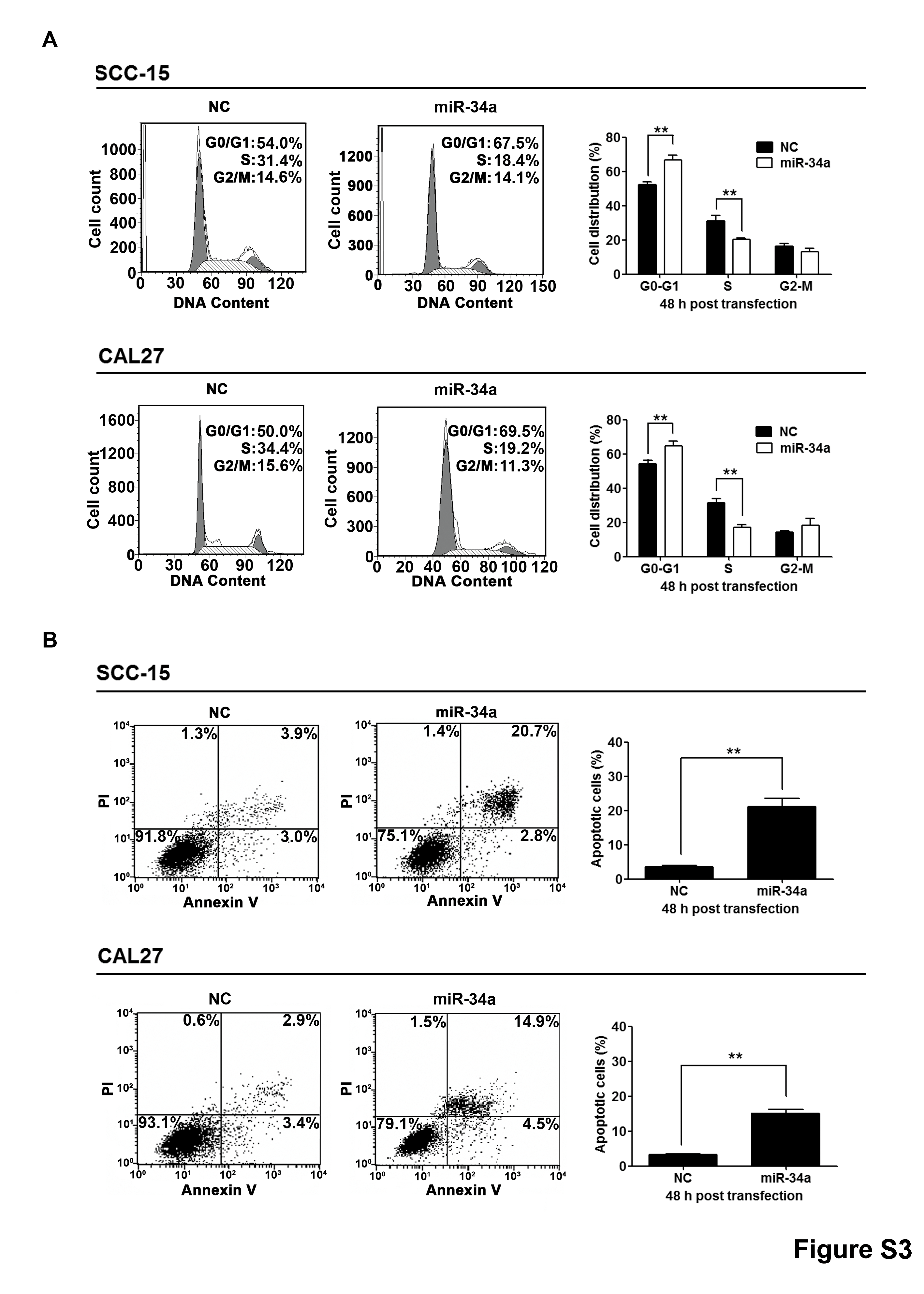

Supplement: Figure S3 — Overexpression of miR-34a inhibited cell cycle progression and promoted cell apoptosis. (A), Inhibition of cell cycle progression by overexpression of miR-34a. SCC-15 and CAL27 cells were transfected with pcDNA3.0, a negative control (NC) or with pcDNA3.0-miR-34a (miR-34a), as indicated. Cells were stained with propidium iodide (PI) at 48 h post-transfection and analyzed with FACS (** P<0.01). (B), Promotion of apoptosis by overexpression of miR-34a. SCC-15 or CAL27 cells were transfected for 48 h as in (A) and apoptotic cells were monitored with FACS after Annexin V and PI staining (** P<0.01). (TIF) [file pone.0108435.s003.tif]

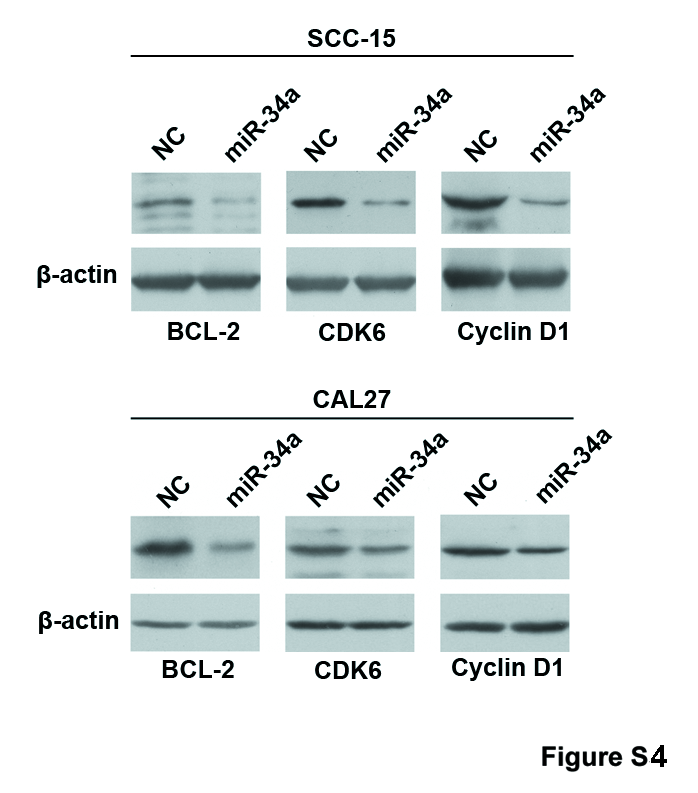

Supplement: Figure S4 — Overexpression of miR-34a decreased the endogenous protein expression of Cyclin D1, CDK6 and Bcl-2 in TSCC cell lines. SCC-15 and CAL27 cells were transfected with pcDNA3.0 as a negative control (NC) or with pcDNA3.0-miR-34a (miR-34a) as indicated. After 48 h, Cyclin D1, CDK6 and Bcl-2 and internal control β-actin were detected by Western blot. (TIF) [file pone.0108435.s004.tif]

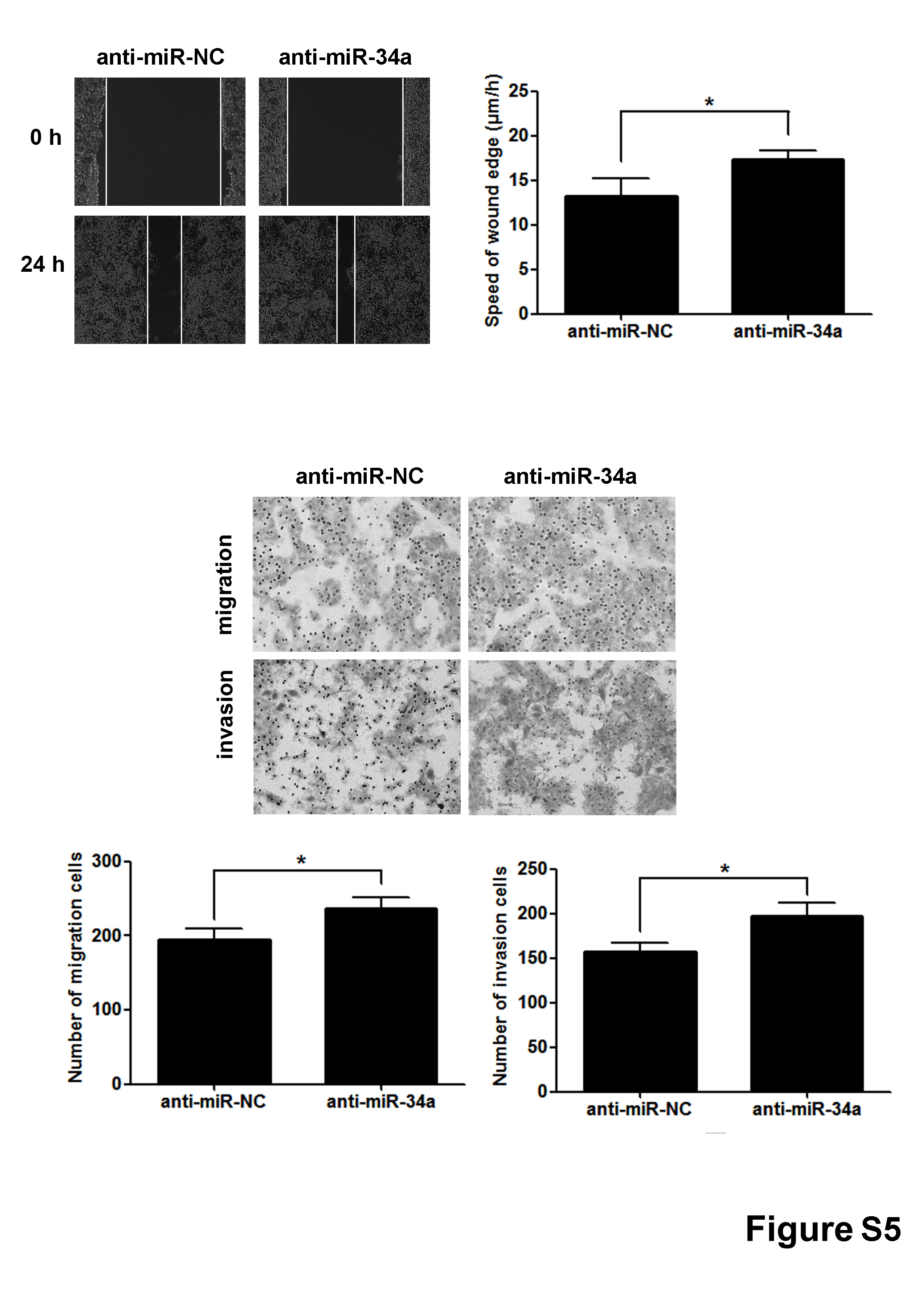

Supplement: Figure S5 — Inhibition of miR-34a in SCC-15 significantly increased cell migration and invasion. Representative photomicrographs of wound healing and transwell assays results for SCC-15 cells transfected with miR-34a inhibitor (anti-miR-34a) or the negative control (anti-miR-NC) (×200 magnification, * P<0.05). (TIF) [file pone.0108435.s005.tif]

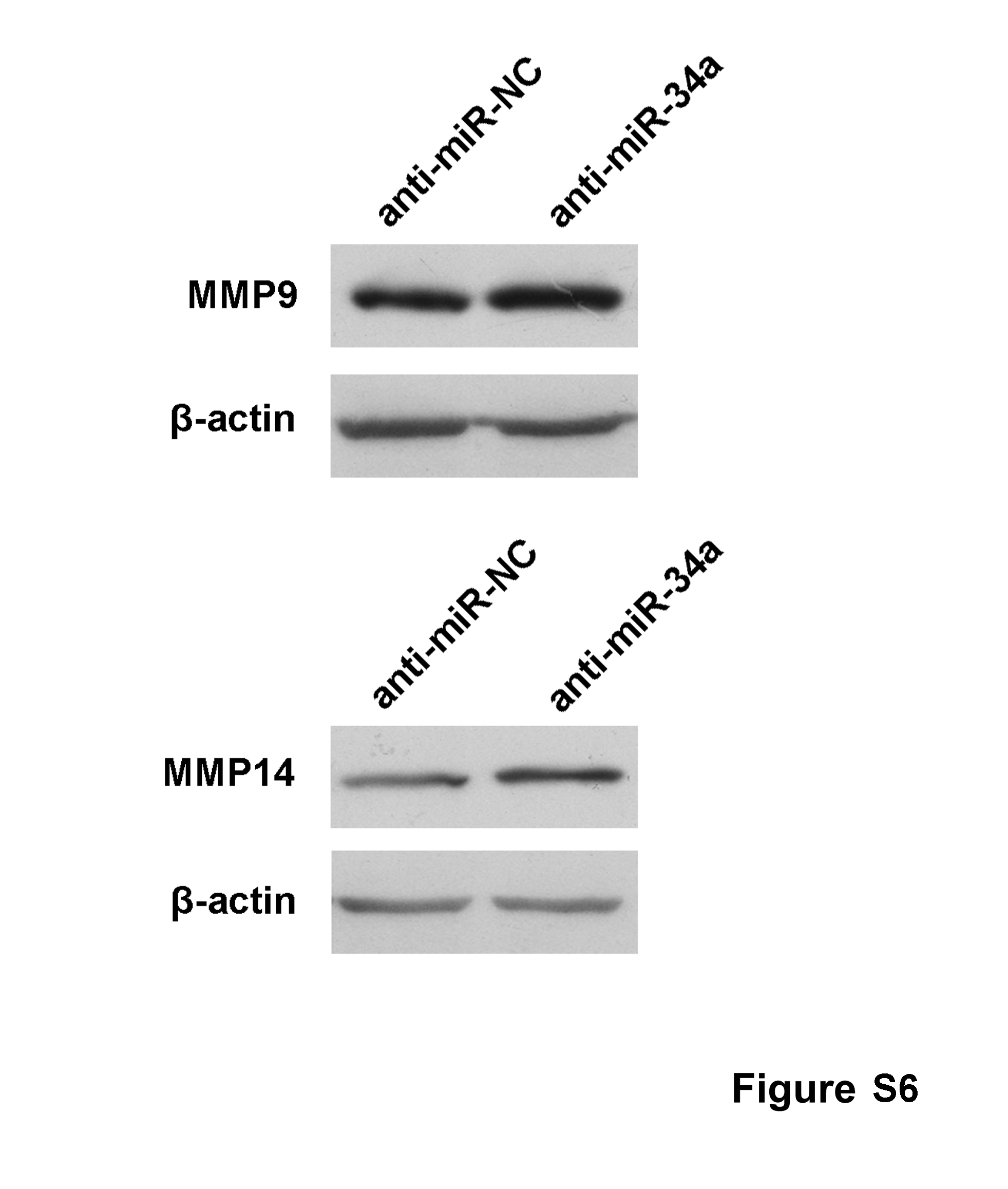

Supplement: Figure S6 — Inhibition of miR-34a in SCC-15 significantly increased protein levels of MMP9 and MMP14. SCC-15 and CAL27 cells were transfected with miR-34a inhibitor (anti-miR-34a) or the negative control (anti-miR-NC) as indicated. After 48 h, MMP9, MMP14 and internal control β-actin were detected by Western blot. (TIF) [file pone.0108435.s006.tif]

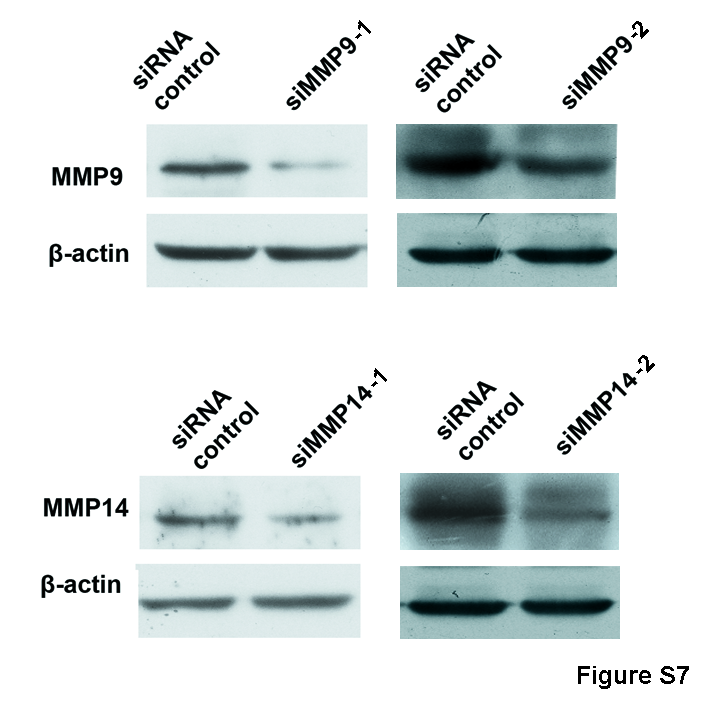

Supplement: Figure S7 — Inhibition of the expression of MMP9 and MMP14 by siRNAs targeting MMP9 and MMP14 transcripts. CAL27 cells were transfected with siRNA control, siMMP9-1, siMMP9-2, siMMP14-1, and siMMP14-2 as indicated. After 24 h, MMP9, MMP14 and internal control β-actin were detected by Western blot. (TIF) [file pone.0108435.s007.tif]

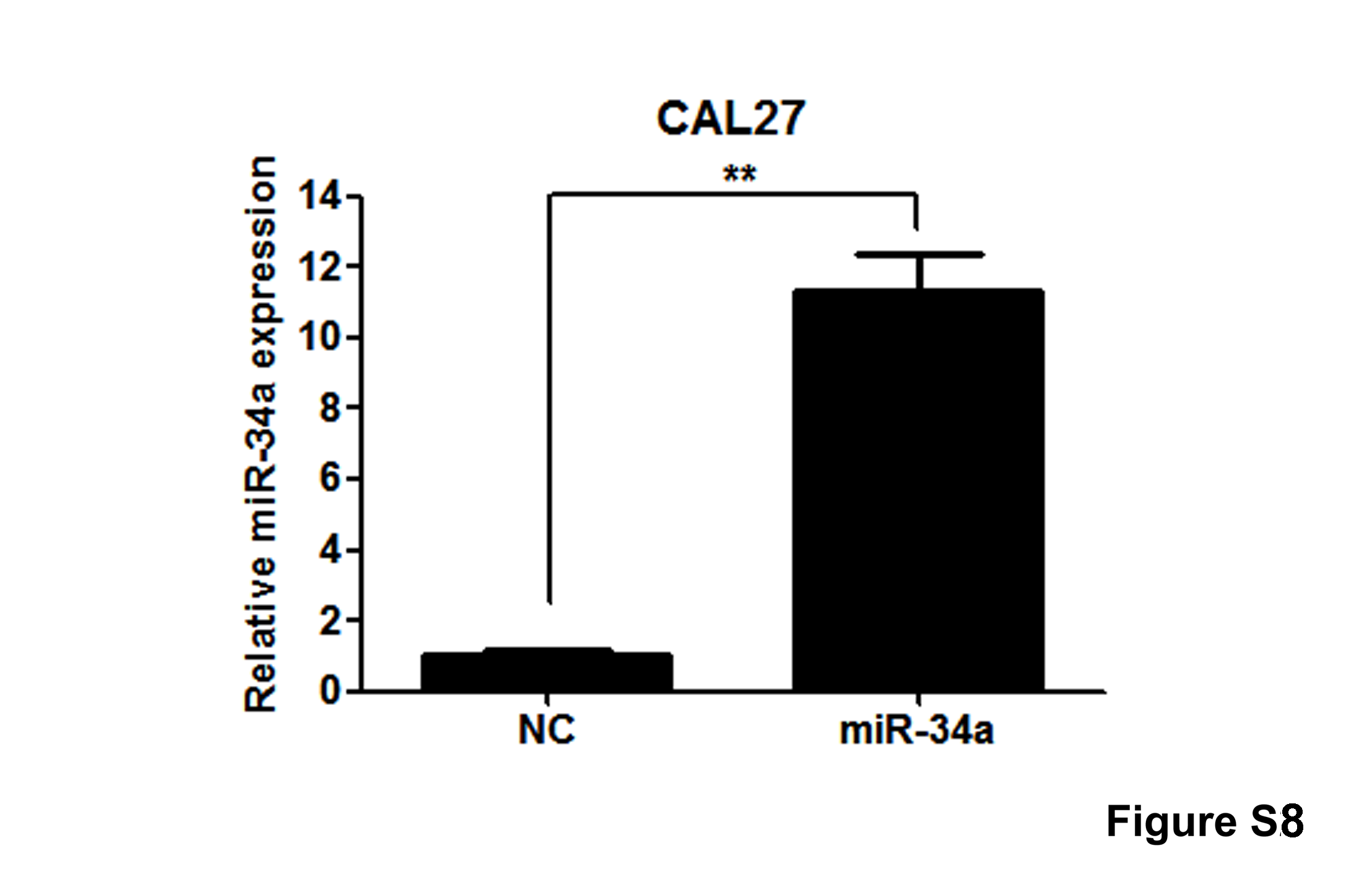

Supplement: Figure S8 — The expression of miR-34a was significantly increased in the miR-34a stably-transfected cells (miR-34a) compared to that stably-transfected with control vectors (NC). Data was presented as mean ±SD (** P<0.01). (TIF) [file pone.0108435.s008.tif]
