# Supplementary material for: miR-34a Inhibits Migration and Invasion of Tongue Squamous Cell Carcinoma via Targeting MMP9 and MMP14
Source: PLoS One. 2014 Sep 30;9(9):e108435. doi: 10.1371/journal.pone.0108435 (PMC4182478; doi:10.1371/journal.pone.0108435)
Supplement: Table S1 — The clinical features of TSCC patients with positive and negative lymph node metastases. (DOC) [file pone.0108435.s009.doc]

**Supplementary Table S1.** The clinical features of TSCC patients with positive and negative lymph node metastases.

| Negative lymph node metastases | | | | | |  | Positive lymph node metastases | | | | | |
| --- | --- | --- | --- | --- | --- | --- | --- | --- | --- | --- | --- | --- |
| NO. | Age/  gender | TNM classification | Differentiation | Final  outcome | Follow-up  period (yr) |  | NO. | Age/  gender | TNM classification | Differentiation | Final  outcome | Follow-up  period (yr) |
| 1 | 40/M | T_3_N_0_M_0_ | Moderate | Alive | 5 |  | 16 | 41/M | T_3_N_0_M_0_ | Moderate | Dead | 2.5 |
| 2 | 53/M | T_3_N_0_M_0_ | Moderate | Alive | 5 |  | 17 | 58/M | T_3_N_0_M_0_ | Moderate | Dead | 0.5 |
| 3 | 42/M | T_3_N_0_M_0_ | Moderate | Alive | 6 |  | 18 | 46/M | T_3_N_0_M_0_ | Moderate | Dead | 3 |
| 4 | 40/F | T_3_N_0_M_0_ | Moderate | Alive | 6.5 |  | 19 | 56/F | T_3_N_0_M_0_ | Moderate | Dead | 3 |
| 5 | 51/M | T_3_N_0_M_0_ | Moderate | Alive | 7 |  | 20 | 50/M | T_3_N_0_M_0_ | Moderate | Dead | 2.5 |
| 6 | 52/F | T_2_N_0_M_0_ | Moderate | Alive | 5 |  | 21 | 59/F | T_2_N_0_M_0_ | Moderate | Dead | 2 |
| 7 | 40/M | T_2_N_0_M_0_ | Moderate | Alive | 6 |  | 22 | 43/M | T_2_N_0_M_0_ | Moderate | Dead | 2 |
| 8 | 53/M | T_2_N_0_M_0_ | Moderate | Alive | 6.5 |  | 23 | 53/M | T_2_N_0_M_0_ | Moderate | Dead | 0.5 |
| 9 | 41/M | T_2_N_0_M_0_ | Moderate | Alive | 6 |  | 24 | 43/M | T_2_N_0_M_0_ | Moderate | Dead | 4 |
| 10 | 69/M | T_2_N_0_M_0_ | Moderate | Alive | 6 |  | 25 | 67/M | T_2_N_0_M_0_ | Moderate | Dead | 4 |
| 11 | 54/F | T_2_N_0_M_0_ | Moderate | Alive | 6.5 |  | 26 | 52/F | T_2_N_0_M_0_ | Moderate | Dead | 2.5 |
| 12 | 62/F | T_2_N_0_M_0_ | Moderate | Alive | 7 |  | 27 | 65/F | T_2_N_0_M_0_ | Moderate | Dead | 3 |
| 13 | 47/M | T_2_N_0_M_0_ | Moderate | Alive | 6 |  | 28 | 48/M | T_2_N_0_M_0_ | Moderate | Dead | 2.5 |
| 14 | 50/M | T_2_N_0_M_0_ | Moderate | Alive | 6.5 |  | 29 | 53/M | T_2_N_0_M_0_ | Moderate | Dead | 2.5 |
| 15 | 61/M | T_2_N_0_M_0_ | Moderate | Alive | 7 |  | 30 | 61/M | T_2_N_0_M_0_ | Moderate | Dead | 1.5 |

Abbreviations: M, male; F, female.
