# Supplementary material for: miR-34a Inhibits Migration and Invasion of Tongue Squamous Cell Carcinoma via Targeting MMP9 and MMP14
Source: PLoS One. 2014 Sep 30;9(9):e108435. doi: 10.1371/journal.pone.0108435 (PMC4182478; doi:10.1371/journal.pone.0108435)
Supplement: Table S2 — Sequences of RNA and DNA Oligonucleotides. (DOC) [file pone.0108435.s010.doc]

**Supplementary Table S2.** Sequences of RNA and DNA Oligonucleotides.

| **Name** | **Sense Strand/Sense Primer (5'-3')** | **Antisense Strand/Antisense Primer (5'-3')** |
| --- | --- | --- |
| **Primers for qRT-PCR** | | |
| RT primer for miR-34a | GTC GTA TCC AGT GCA GGG TCC GAG GTA TTC GCA CTG GAT ACG ACG CCA AT | |
| miR-34a | CGT AGC AGC ACA GAA AT | GTG CAG GGT CCG AGG T |
| U6 | CTC GCT TCG GCA GCA CA | AAC GCT TCA CGA ATT TGC GT |
| MMP9 | GCC CGA CCC GAG CTG ACT C | TTC AGG GCG AGG ACC ATA GAG G |
| MMP14 | TCG GCC CAA AGC AGC AGC TTC | CTT CAT GGT GTC TGC ATC AGC |
| β-actin | CGG GAA ATC GTG CGT GAC | CAG GCA GCT CGT AGC TCT T |
|  |  |  |
| **miR-34a inhibitor and negative control sequence** | | |
| anti-miR-34a | ACA ACC AGC UAA GAC ACU GCC A | |
| anti-miR-NC | UCU ACU CUU UCU AGG AGG UUG UGA | |
|  |  |  |
| **siRNA Duplexes** | | |
| siMMP9-1 | CAU CAC CUA UUG GAU CCA ATT | UUG GAU CCA AUA GGU GAU GTT |
| siMMP9-2 | GGA ACC AGC UGU AUU UGU UTT | AAC AAA UAC AGC UGG UUC CTT |
| siMMP14-1 | CCU ACG AGA GGA AGG AUG GCA AAU UTT | AAU UUG CCA UCC UUC CUC UCG UAG GTT |
| siMMP14-2 | CAG GCA AAG CUG AUG CAG ATT | UCU GCA UCA GCU UUG CCU GTT |
| siRNA control | UUC UCC GAA CGU GUC ACG UTT | ACG UGA CAC GUU CGG AGA ATT |
|  |  |  |
| **The probe sequence used for in situ hybridization** | | |
| miR-34a | ACA ACC AGC TAA GAC ACT GCC A | |
| Scramble-miR | GTG TAA CAC GTC TAT ACG CCC A | |
